# Gating and Regulatory Mechanisms of TMEM16 Ion Channels and Scramblases

**DOI:** 10.3389/fphys.2021.787773

**Published:** 2021-11-19

**Authors:** Son C. Le, Pengfei Liang, Augustus J. Lowry, Huanghe Yang

**Affiliations:** ^1^Department of Biochemistry, Duke University Medical Center, Durham, NC, United States; ^2^Department of Neurobiology, Duke University Medical Center, Durham, NC, United States

**Keywords:** TMEM16, Anoctamin, CaCC, lipid scramblase, phosphatidylserine, PIP2, pH, calcium

## Abstract

The transmembrane protein 16 (TMEM16) family consists of Ca^2+^-activated ion channels and Ca^2+^-activated phospholipid scramblases (CaPLSases) that passively flip-flop phospholipids between the two leaflets of the membrane bilayer. Owing to their diverse functions, TMEM16 proteins have been implicated in various human diseases, including asthma, cancer, bleeding disorders, muscular dystrophy, arthritis, epilepsy, dystonia, ataxia, and viral infection. To understand TMEM16 proteins in health and disease, it is critical to decipher their molecular mechanisms of activation gating and regulation. Structural, biophysical, and computational characterizations over the past decade have greatly advanced the molecular understanding of TMEM16 proteins. In this review, we summarize major structural features of the TMEM16 proteins with a focus on regulatory mechanisms and gating.

## Introduction

Since the elegant experiments that led to the discoveries of TMEM16A/ANO1 and TMEM16B/ANO2 as the long-sought-after Ca^2+^-activated Cl^−^ channels (CaCCs) in 2008 (Caputo et al., 2008; Schroeder et al., 2008; Yang et al., 2008), substantial progress has been made to understand the biology of this unique family of transmembrane proteins. Numerous studies confirmed that TMEM16A and TMEM16B are responsible for the endogenous CaCC currents observed in various cell types (Bader et al., 1982; Miledi, 1982; Barish, 1983). More excitingly, new findings uncovered their novel physiological and pathological functions, including smooth muscle contraction, trans-epithelial fluid transport, secretion, tumor progression, sensory transduction, mood control, and motor learning (Hartzell et al., 2005; Duran and Hartzell, 2011; Pedemonte and Galietta, 2014; Oh and Jung, 2016; Whitlock and Hartzell, 2016a; Zhang et al., 2017; Crottes and Jan, 2019).

Among the most striking findings in TMEM16 research is that, unlike initial predictions, the remaining family members are likely not CaCCs. Instead, the majority of the TMEM16 family members characterized thus far are Ca^2+^-activated phospholipid scramblases (CaPLSases), which can translocate phospholipids down their chemical gradients in a relatively non-selective fashion. As passive phospholipid transporters, TMEM16 CaPLSases can efficiently translocate phospholipids at high speed (4.5×10^4^ phospholipids per second for TMEM16F; Watanabe et al., 2018). Therefore, activation of TMEM16 CaPLSases leads to rapid collapse of membrane phospholipid asymmetry, which can trigger a plethora of cellular responses and physiological functions, such as blood coagulation (Suzuki et al., 2010; Yang et al., 2012), microparticle release (Fujii et al., 2015), membrane repair (Wu et al., 2020), sheddase activation (Sommer et al., 2016; Veit et al., 2018; Bleibaum et al., 2019), endosomal sorting (Petkovic et al., 2020), cell–cell fusion (Griffin et al., 2016; Whitlock et al., 2018; Zhang et al., 2020; Braga et al., 2021), and viral infection (Bevers and Williamson, 2016; Zaitseva et al., 2017; Younan et al., 2018). While the list of new biological functions of TMEM16 CaPLSases and CaCCs keeps growing, their importance in human health and disease has become apparent, as malfunctions in TMEM16 proteins have been implicated in human diseases, including asthma, cancer, bleeding disorders, muscular dystrophy, arthritis, epilepsy, dystonia, and ataxia (Duran and Hartzell, 2011; Pedemonte and Galietta, 2014; Oh and Jung, 2016; Crottes and Jan, 2019). To target TMEM16 proteins and treat TMEM16-related diseases, it is critical to have a comprehensive understanding of these novel proteins at the molecular level.

Structural, functional, and computational characterizations of TMEM16 proteins have provided an in-depth understanding of the mechanisms of permeation, activation, and regulation. Given the space limit of this review, we first briefly summarize the key structural features of TMEM16F CaCCs and CaPLSases and then focus on discussing the molecular mechanism of Ca^2+^-dependent gating, and how an allosteric Ca^2+^ binding site, phosphatidylinositol-(4,5)-bisphosphate [or PI(4,5)P_2_], and pH regulate TMEM16 Ca^2+^-dependent gating. This is by no means a comprehensive review of TMEM16 structure and function. The readers are encouraged to refer to the excellent reviews of the biophysics (Brunner et al., 2016; Whitlock and Hartzell, 2016b; Falzone et al., 2018; Kalienkova et al., 2021; Le and Yang, 2021) and physiology of TMEM16 proteins (Hartzell et al., 2005; Duran and Hartzell, 2011; Pedemonte and Galietta, 2014; Oh and Jung, 2016; Whitlock and Hartzell, 2016a).

## Overall Architecture of Tmem16 Proteins

**Figure 1:**
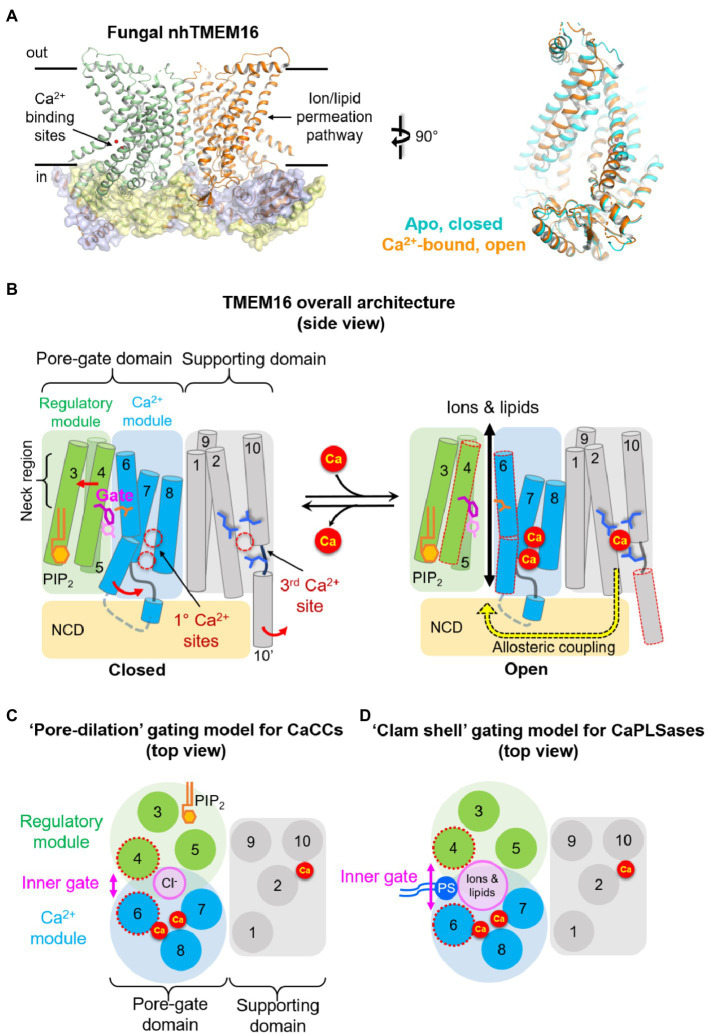
Architecture of TMEM16 proteins. **(A)** Left: X-ray structure of the fungal nhTMEM16 bound to Ca^2+^ (PDB: 4WIS). Right: comparisons of the permeation pathway from cryo-EM structures of nhTMEM16 in an apo, closed state (cyan, PDB: 6QM4) and Ca^2+^-bound, open state (orange, PDB: 6QM9). **(B)** A simplified cartoon showing the overall architecture (side view) and the “modular design” model of TMEM16 proteins. Three sidechains in the middle of the pore represent the inner activation gate residues (F518, Y563, and I612) of TMEM16F CaPLSase. The putative conformational changes induced by Ca^2+^ binding and subsequent activation gate opening are shown on the right. The neck region refers to the narrowest region of the permeation pathway. NCD, N-terminal cytosolic domain; PIP_2_, PI(4,5)P_2._ **(C)** A top view at the level of the inner activation gate showing the “pore-dilation” gating model for TMEM16 CaCCs. According to this model, Ca^2+^-induced conformational changes dilate the permeation pore without separating the TM4/TM6 interface. In this way, only Cl^−^ ions but not phospholipids permeate through the protein-enclosed activation gate. **(D)** A top view at the level of the inner activation gate showing the “clam-shell” gating model for TMEM16 CaPLSases. According to this model, Ca^2+^-induced conformational changes lead to the separation of TM4 and TM6 at the neck region, resulting in a semi-open pore that faces the lipid core of the membrane. This clam shell-like opening enables phospholipid headgroups to access and subsequently permeate through the pore.

The first glimpse into the atomic structure of TMEM16 proteins came from the X-ray structures of a fungal TMEM16 homolog from *Nectria haematococca* (or nhTMEM16, Figure 1A Left; Brunner et al., 2014), which functions as a CaPLSase and likely also a Ca^2+^-activated nonselective channel (Lee et al., 2016). Subsequent structural analyses of the fungal afTMEM16, mouse TMEM16A, mouse TMEM16F, and human TMEM16K all revealed their highly conserved architecture (Brunner et al., 2014; Dang et al., 2017; Paulino et al., 2017a,b; Alvadia et al., 2019; Falzone et al., 2019; Feng et al., 2019; Kalienkova et al., 2019). Similar to ClC Cl^−^ channels and Cl^−^/H^+^ exchangers (Miller, 2006), a functional TMEM16 protein is a dimer with a double-barreled architecture, in which an independent permeation pore resides in each subunit. The double-barreled architecture was functionally validated by electrophysiological characterizations of TMEM16A concatemers, where each monomer possessed different Ca^2+^ sensitivities or ion selectivities (Jeng et al., 2016; Lim et al., 2016).

**Figure 2:**
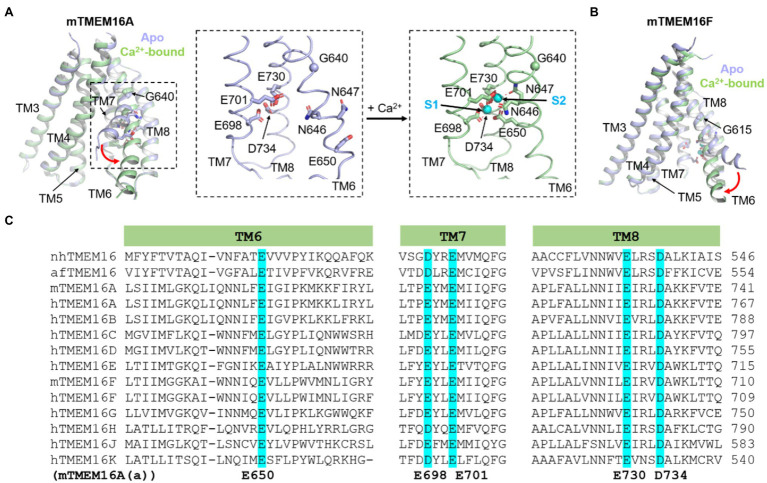
Ca^2+^-dependent activation of TMEM16A CaCC and TMEM16F CaPLSase. **(A)** Ca^2+^-induced conformational changes of TMEM16A. Insets show close-up views of the Ca^2+^ binding sites of TMEM16A. S1 and S2 refer the lower and upper bound Ca^2+^ ions, respectively. **(B)** Ca^2+^-induced conformational changes of TMEM16F. The Ca^2+^-free (apo) structures are shown in light blue, and the Ca^2+^-bound structures are in light green. PDB codes are 5OYG and 5OYB for apo and Ca^2+^-bound mouse TMEM16A, respectively, and are 6QPB and 6QP6 for apo and Ca^2+^-bound mouse TMEM16F, respectively. Only TMs 3–8 are shown for clarity. **(C)** Protein sequence alignment of the fungal nhTMEM16 and afTMEM16 and human (h) and murine (m) TMEM16A-K showing the highly conserved Ca^2+^ binding residues in TM6, 7 and 8 (highlighted in cyan). Numbering of TMEM16A’s Ca^2+^ binding residues is based on the (a) isoform.

Different from the initial prediction of an 8-transmembrane (TM) topology, we now know that each TMEM16 monomer consists of 10 TM segments preceded by a long N-terminal cytosolic domain (NCD) and followed by a short C-terminal extension of TM10 (Figure 1B). TM7 and TM8 do not completely traverse the membrane, which, together with TM6, form two highly conserved Ca^2+^ binding sites (Figures 1B, 2). The anion permeation pathway of the TMEM16A is shaped like an asymmetric hourglass and is formed by numerous hydrophilic and nonpolar residues from TMs 3–7. The so-called hydrophilic cavity has been shown to form a non-selective permeation pathway for not only ions in the TMEM16 channels, but also phospholipids in the scramblases (Brunner et al., 2014; Dang et al., 2017; Jiang et al., 2017; Paulino et al., 2017a,b; Lee et al., 2018; Bushell et al., 2019; Falzone et al., 2019; Le et al., 2019b). Notably, in the fungal nhTMEM16 and afTMEM16 as well as the human TMEM16K structures, the hydrophilic cavity has been captured in an “open” conformation in which the peripheral TM4 and TM6 are physically separated, exposing the hydrophilic cavity to the lipid environment (Figure 1A Right; Falzone et al., 2018; Kalienkova et al., 2021). This putative “open” lipid-conducting state supports the notion that TMEM16 scramblases catalyze lipid translocation *via* a “credit card” model previously proposed for phospholipid flippases (Pomorski and Menon, 2006). This model implies that the headgroups of permeating phospholipids may slide along the hydrophilic groove of TMEM16 scramblases, while their acyl tails remain in the hydrophobic lipid environment, a hypothesis that has been supported by extensive structural, functional, and molecular dynamics (MD) studies (Brunner et al., 2014; Bethel and Grabe, 2016; Jiang et al., 2017; Lee et al., 2018; Bushell et al., 2019; Kalienkova et al., 2019; Le et al., 2019b). For dual function ion channel/scramblases, ions may permeate adjacent to lipid headgroups through a proteolipid pore (Whitlock and Hartzell, 2016b). In support of this idea, a recent computational study suggested that the ion permeation pathway in the fungal nhTMEM16 and human TMEM16K is partially lined by ordered lipid headgroups (Kostritskii and Machtens, 2021). The lipid headgroup identity, pore-lining residues, and membrane voltage all exert appreciable effects on ion permeation and selectivity (Kostritskii and Machtens, 2021). By contrast, all current Ca^2+^-bound structures of the TMEM16A CaCC and the dual function TMEM16F ion channel/scramblase paradoxically adopt tightly closed permeation pathways that are too narrow to allow the passage of ions or lipids (Dang et al., 2017; Paulino et al., 2017a; Alvadia et al., 2019; Feng et al., 2019; Figures 2A,B). The reason for these structural observations remains elusive and requires future investigation.

Based on structural, functional, and computational evidence of Ca^2+^-dependent activation and PI(4,5)P_2_-dependent regulation (see in the next sections), we recently proposed a modular model of TMEM16 proteins to simplify the complex TMEM16 architecture (Figures 1B–D; Le et al., 2019a). According to this model, a TMEM16 monomer can be divided into several structurally and functionally distinct domains and modules. Besides the NCD, the transmembrane region can be divided into two domains: the pore-gate domain (PGD) and the supporting domain. Consisting of TMs 3–8, the PGD not only forms the permeation pathway for ions and phospholipids, but also harbors the highly conserved primary Ca^2+^ binding sites (Figures 2A,C) and, in the case of TMEM16A, a putative PI(4,5)P_2_ binding site (Figures 1B,C). As Ca^2+^ binding and PI(4,5)P_2_ binding are structurally and functionally segregated, the PGD of TMEM16A can be further divided into two modules (Le et al., 2019a). The Ca^2+^ module consists of TMs 6–8 and is responsible for binding Ca^2+^ and initiating Ca^2+^-dependent activation. The regulatory module (TMs 3–5) forms the other half of the PGD, which works synergistically with the Ca^2+^ module to facilitate TMEM16 gating and permeation. In TMEM16A, PI(4,5)P_2_ binding to the regulatory module stabilizes the open state and prevents the PGD from collapsing and entering the desensitized state. TMs 1, 2, 9, and 10, on the other hand, form the supporting domain. As will be discussed below, the supporting domain contains a conserved third Ca^2+^ binding site that is allosterically coupled to the PGD (Figure 1B). The supporting domain also helps establish the dimer interface within the membrane through inter-subunit interactions between the extracellular regions of TM10. The dimer interface creates two large hydrophobic cavities, or dimer cavities, along the central axis of all TMEM16 proteins. In addition to the TM10 interactions, the fungal nhTMEM16 and afTMEM16 as well as the human TMEM16K adopt a “domain-swapped” organization in which their C-terminal ends have extensive inter-subunit interactions with the NCD of the adjacent protomer. Interestingly, “domain-swapping” is not observed in the TMEM16A and TMEM16 F structures. Beyond facilitating dimer formation, supporting domain interactions potentially serve to stabilize the PGD.

It is worth noting that four conserved disulfide bonds constrain the relatively long extracellular loops connecting TM1-2, 3–4, 5–6, and 9–10 in most mammalian TMEM16 proteins, including TMEM16A and TMEM16F. Disruption of these disulfide bonds leads to dysfunctional channels (Yu et al., 2012), suggesting that the stability of the extracellular loops is important for TMEM16A and TMEM16F activity. Interestingly, the extracellular loops of the fungal nhTMEM16 and afTMEM16 and the endoplasmic reticulum (ER)-resident human TMEM16K scramblase are significantly shorter and lack disulfide bonds (Brunner et al., 2014; Bushell et al., 2019; Falzone et al., 2019). Since these TMEM16 proteins still function as CaPLSases and Ca^2+^-activated nonselective ion channels, the long extracellular loops seem dispensable for ion and lipid transport functions. The precise functions of the extracellular loops are unclear and require future investigation.

In summary, structure/function studies have elucidated many of the defining elements of the TMEM16 family. These elements may be conserved in the evolutionarily related osmo- and mechano-sensing OSCA/TMEM63A (Murthy et al., 2018) and TMC channels (Hahn et al., 2009; Ballesteros et al., 2018; Medrano-Soto et al., 2018). To simplify the growing complexity, we propose a modular design of the TMEM16 proteins (Figures 1B,C), which may also facilitate the understanding of the molecular mechanisms of related proteins.

## Ca^2+^-Dependent Activation Of Tmem16 Proteins

All mammalian TMEM16 ion channels and lipid scramblases require the binding of intracellular Ca^2+^ for activation, albeit at different levels of potency. The TMEM16A and TMEM16B CaCCs are synergistically gated by intracellular Ca^2+^ and membrane voltages. TMEM16A is highly sensitive to Ca^2+^ with an estimated EC_50_ of 0.4 to 1μM at positive membrane potentials or 0.7 to 6μM at negative membrane potentials (Yang et al., 2008; Ferrera et al., 2009; Xiao et al., 2011; Yu et al., 2012; Brunner et al., 2014; Ni et al., 2014; Tien et al., 2014; Lim et al., 2016; Cruz-Rangel et al., 2017; Le et al., 2019a). Despite their similarity, TMEM16B displays a lower Ca^2+^ sensitivity with an estimated EC_50_ of around 1.2 to 3.3μM at positive membrane potentials and 1.8 to 4.9μM at negative potentials (Pifferi et al., 2009; Stephan et al., 2009; Cenedese et al., 2012; Adomaviciene et al., 2013; Pifferi, 2017). One notable feature is that TMEM16A- and TMEM16B-mediated currents are outward rectifying under the low open probability and display time-dependent activation and deactivation kinetics (Caputo et al., 2008; Yang et al., 2008; Pifferi et al., 2009; Stephan et al., 2009). However, these channels are no longer time- and voltage-dependent when they are fully opened by saturating Ca^2+^.

The dual function TMEM16F ion channel and phospholipid scramblase is less sensitive to Ca^2+^. The estimated EC_50_ values range from 3.4 to 105μM, depending on the configuration and ionic conditions of the patch clamp recording (Yang et al., 2012; Grubb et al., 2013; Shimizu et al., 2013; Scudieri et al., 2015; Feng et al., 2019; Le et al., 2019b; Nguyen et al., 2019; Ye et al., 2019). The Ca^2+^ sensitivity for TMEM16F scrambling activity has not been accurately measured. However, based on the co-occurrence of TMEM16F current and scramblase activity recorded using patch clamp-lipid scramblase fluorometry (PCLSF) assay (Yu et al., 2015; Liang and Yang, 2021), it is expected that the Ca^2+^ sensitivity for TMEM16F CaPLSase activity is comparable to the Ca^2+^ sensitivity for channel activity. TMEM16F-mediated ionic conductance is elicited by the synergistic activation of membrane depolarization and Ca^2+^ binding (Yang et al., 2012). Unlike TMEM16A and TMEM16B, the TMEM16F channel always requires membrane depolarization for activation and its current remains strongly outward rectifying even at high Ca^2+^ concentrations. It is yet unknown whether membrane voltage can promote CaPLSase activity.

Mutagenesis studies on TMEM16A CaCC successfully identified five highly conserved acidic residues as putative Ca^2+^ binding residues, including E650 on TM6, E698 and E701 on TM7, E730 and D734 on TM8 (Yu et al., 2012; Tien et al., 2014; Figures 2A,C, numbering based on the TMEM16A(a) isoform lacking the EAVK segment). Neutralizing mutations (to alanine or glutamine) strongly reduce the Ca^2+^ sensitivity of TMEM16A from the sub-micromolar range to the millimolar range. Subsequent structural and functional studies not only validated these electrophysiological findings but also revealed three additional asparagine residues (N646 and N647 of TM6 and N726 of TM8) as additional Ca^2+^ coordinates (Brunner et al., 2014; Dang et al., 2017; Paulino et al., 2017a). Within each TMEM16 monomer, the Ca^2+^ binding residues cluster together and form two highly conserved Ca^2+^ binding sites, herein referred to as the primary Ca^2+^ sites (Figures 1B, 2). The highly conserved primary Ca^2+^ binding sites among different TMEM16 homologs suggest that these evolutionarily conserved proteins maintain a similar activation mechanism.

The primary TMEM16 Ca^2+^ binding sites have several unique features (Figures 1B, 2). First, the Ca^2+^ binding residues reside within the membrane electrical field, which is in excellent agreement with a previous prediction (Arreola et al., 1996). The membrane location of the Ca^2+^ binding sites in TMEM16 proteins may partially contribute to their weak voltage-dependent Ca^2+^ activation (Hartzell et al., 2005; Pifferi et al., 2009; Xiao et al., 2011; Yang et al., 2012), as Ca^2+^ ions need to travel within the membrane electric field to reach the binding sites. Second, the primary Ca^2+^ binding sites are located near the ion/lipid permeation pathway. Such proximity between the Ca^2+^ binding sites and the activation gates implies that TMEM16 proteins can efficiently transmit Ca^2+^ binding energy to operate their activation gates.

Structural and functional studies have shown that Ca^2+^-induced TM6 conformational changes are critical for Ca^2+^-dependent activation of both TMEM16 ion channels and scramblases (Figures 1, 2; Dang et al., 2017; Paulino et al., 2017a; Peters et al., 2018; Alvadia et al., 2019; Feng et al., 2019). Structural studies of the TMEM16A CaCC showed that in the absence of Ca^2+^, TM6 adopts an alpha-helical conformation with a kink at G640 (Figures 2A,C; Dang et al., 2017; Paulino et al., 2017a). This kink causes the C-terminal segment of TM6 to swing away from TM7 and TM8, thereby rendering the negatively charged Ca^2+^ binding residues accessible to the cytosol. The highly electronegative environment created by the apo Ca^2+^ binding sites also serves to impede Cl^−^ entry from the intracellular side (Paulino et al., 2017b; Lam and Dutzler, 2018). It was suggested that Ca^2+^ ions first bind to and neutralize the four highly acidic residues from TM7 and TM8, providing an attractive environment that allows TM6 to move toward TM7 and TM8 by interacting with the bound Ca^2+^ ions *via* N647 and E650. During this process, TM6 rotates around the G640 hinge because of the interactions between N647, E650, and the two bound Ca^2+^, subsequently leading to the formation of a π-helix (Figure 2A). Superimposing the Ca^2+^-bound and Ca^2+^-free structures reveals that Ca^2+^ binding leads to partial widening of the central constriction site in TMEM16A, though, paradoxically, the permeation pathway is still too narrow for anion passage. Supporting the functional importance of TM6 in TMEM16A gating, several mutations on TM6 such as I637A, I637K, G640A/P, Q645A, and P654A were shown to alter the channel’s Ca^2+^ sensitivity (Dang et al., 2017; Paulino et al., 2017a; Lam and Dutzler, 2018; Peters et al., 2018; Le et al., 2019b). These mutations likely shift the equilibrium of TM6 to favor either the open conductive state (G640A/P, I637A/K, and Q645A) or the closed non-conductive state (P654A). A recent computational study further supports the importance of TM6 conformational changes in Ca^2+^-dependent gating of TMEM16A (Shi et al., 2021). Based on MD simulations, the authors concluded that separation of TM6 and TM4 may lead to expansion of the ion permeation pore and consequently the opening of the channel. This is consistent with the “pore-dilation” model (Figure 1C) derived from functional tests (Le et al., 2019b).

Conformational changes of TM6 also seem critical for the gating of the TMEM16F ion channel/scramblase, albeit *via* an opposite movement of the cytosolic end of TM6 compared to TMEM16A TM6 (Figure 2). However, analogous to TMEM16A, binding of two Ca^2+^ ions to N620, N621, and E624 of TM6, E667 and E670 of TM7, and E699 and D703 of TM8 neutralizes the Ca^2+^ binding sites and allows TM6 to approach TM7 and TM8 *via* a rigid body movement around G615, equivalent to TMEM16A’s G640 (Figure 2). Because of a missing residue near the G615 hinge (Figure 2C), Ca^2+^ binding does not result in partial unwinding of TM6 and hence the π-helix does not form in TMEM16F (Alvadia et al., 2019; Feng et al., 2019). A similar transition from a bent to straight conformation of TM6 was also observed in the structures of TMEM16F with zero or one Ca^2+^ bound, respectively (Feng et al., 2019). It is worth noting that while the fungal afTMEM16 and nhTMEM16 homologs lack a glycine hinge, TM6 also undergoes a similar swinging movement around the equivalent region upon Ca^2+^ binding (Falzone et al., 2019; Kalienkova et al., 2019). These observations further illuminate the conserved gating mechanism shared among TMEM16 ion channels and scramblases.

While Ca^2+^-induced conformational changes in TM6 were unambiguously shown to be critical for the gating of TMEM16 ion channels and scramblases, recent studies on TMEM16A (Tak et al., 2019) and TMEM16F (Roh et al., 2021) proposed another interesting Ca^2+^-dependent gating. Tak et al. (2019) suggested that the TMEM16A CaCC harbors an EF-hand-like domain consisting of a cluster of acidic residues (TMEM16A D285 to D297) that could serve as a reservoir for Ca^2+^ binding before being transferred to the primary sites in TMs 6–8 for subsequent activation. Neutralization of these acidic residues reduces both TMEM16A’s Ca^2+^ and voltage sensitivity. While TMEM16F does not appear to have such an EF-hand-like domain, Roh et al. (2021) showed that neutralizing acidic residues in the equivalent N-terminal domain of TMEM16F reduces its Ca^2+^ sensitivity, consistent with the importance of this acidic Ca^2+^ reservoir in channel gating. Furthermore, the N-terminal Ca^2+^ reservoir in TMEM16F has less acidic residues compared to that of TMEM16A and contains additional basic residues. Replacing the N-terminal Ca^2+^ reservoir of TMEM16F with the equivalent EF-hand-like N-terminal domain of TMEM16A markedly enhances TMEM16F’s Ca^2+^ sensitivity, suggesting that the differences in electronegativity at this region may contribute to determining Ca^2+^-dependent gating in TMEM16 proteins (Tak et al., 2019; Roh et al., 2021).

Another intriguing phenomenon about TMEM16F Ca^2+^-dependent activation is the long (~5–10min) delay after establishing the whole-cell patch clamp configuration (Grubb et al., 2013; Shimizu et al., 2013; Scudieri et al., 2015; Yu et al., 2015; Lin et al., 2018; Liang and Yang, 2021; Stabilini et al., 2021). This delay persists even when the pipette solution contains 100–200μM Ca^2+^. Therefore, the delay cannot be simply explained by the relatively low Ca^2+^ sensitivity of TMEM16F, which may require prolonged diffusion time for intracellular Ca^2+^ to reach the threshold concentration to activate TMEM16F. Paradoxically, TMEM16F current can be instantaneously activated without delay under inside-out configuration (Yang et al., 2012; Lin et al., 2018; Liang and Yang, 2021). It seems apparent that some intracellular factors might be responsible for the patch configuration-dependent discrepancy on TMEM16F activation. Although the detailed mechanisms are still unclear, a recent study provided important clues (Lin et al., 2018). The authors found that disrupting the actin cytoskeleton with cytochalasin-D (cytoD) significantly shortens the delay and accelerates TMEM16F activation. Analogously, the actin filament-stabilizing agents phalloidin and jasplakinolide inhibit TMEM16F current activation. These results suggest that the actin cytoskeleton may negatively regulate TMEM16F ion channel activity under the whole-cell configuration. Interesting, the authors also showed that intracellular magnesium ATP but not sodium ATP further prolongs the delay for TMEM16F current activation. How these intracellular factors affect TMEM16F current activation and if they also affect TMEM16F lipid scrambling activity warrant further investigations.

## Tmem16 Inner Activation Gate

Structural, functional, and computational studies have demonstrated a crucial role for pore-lining TM6 residues in gating of both TMEM16 channels and TMEM16 scramblases. However, a comprehensive understanding of their gating mechanisms requires the identification of the physical activation gate that opens and closes to control ion and phospholipid permeation in response to Ca^2+^ binding. Such activation gates have been proposed for both TMEM16A and TMEM16F (Le et al., 2019b; Lam et al., 2021). Using MD simulations and an optimized lipid scrambling assay, three bulky and hydrophobic residues–F518 in TM4, Y563 in TM5, and I612 in TM6, were identified as the major constituents of the scramblase inner steric activation gate in TMEM16F (Figure 1B; Le et al., 2019b). Removing steric hindrance *via* alanine substitutions of these residues leads to constitutively active TMEM16F scramblases, whereas substitution with leucine or a bulky tryptophan strongly impairs TMEM16F scrambling activity following Ca^2+^ stimulation. On the other hand, mutating the inner gate with polar or charged residues greatly enhances TMEM16F lipid scrambling and ion channel activities. Most of these mutations require culturing the transfected cells in Ca^2+^-free media to suppress TMEM16F gain-of-function (GOF)-induced cytotoxicity, suggesting that basal Ca^2+^ activity is sufficient to open the inner activation gate. Remarkably, F518K and Y563K result in constitutively active TMEM16F scramblases even when the primary Ca^2+^ binding sites are destroyed. More strikingly, the TMEM16A L543K mutation, equivalent to TMEM16F F518K, converts the TMEM16A CaCC into a GOF phospholipid scramblase (Le et al., 2019b). Based on these functional observations and various TMEM16 scramblases captured in different conformations (Alvadia et al., 2019; Falzone et al., 2019; Feng et al., 2019; Kalienkova et al., 2019), a “clam-shell” model was proposed to describe the Ca^2+^-dependent gating of the TMEM16 phospholipid permeation pathway (Le et al., 2019b; Figure 1D). According to this model, Ca^2+^-induced conformational changes at the primary Ca^2+^ binding sites interrupt the interactions between TM4 and TM6 in the neck region, leading to the separation of TM4 and TM6. This clam-shell-type opening exposes the hydrophilic interior of the permeation pathway to the hydrophobic phase of the membrane, thereby allowing phospholipid headgroups to gain access and scramble (Figures 1B,D). Clam-shell opening also enables ion permeation through the proteolipid pore. Replacing the bulky, hydrophobic residues at the inner activation gate with smaller, polar, or charged amino acids weakens the interactions between TM4 and TM6, leading to enhanced permeation or a constitutively open permeation pathway for both lipids and ions.

As Cl^−^ permeation through CaCC requires an enclosed protein environment, it is conceivable that TMEM16A gating may not follow the “clam-shell” gating model of the TMEM16 scramblases. Instead, Ca^2+^-induced conformational changes only appear to dilate the central pore of TMEM16A, allowing tight control of Cl^−^ permeation (Dang et al., 2017; Paulino et al., 2017a; Le et al., 2019b; Shi et al., 2021; Figure 1C). The hydrophobic residues L543, I546, I547, and I637 (L547, I550, I551, and I641 in the (ac) isoform) at the equivalent locations to the TMEM16F inner gate residues likely form the hydrophobic gate to control TMEM16A Cl^−^ permeation (Le et al., 2019b; Lam et al., 2021) as evidenced by alanine and lysine mutations promoting TMEM16A activation. Interestingly, L543K enables TMEM16A activation in the absence of Ca^2+^ and reduces its anion selectivity, in addition to converting TMEM16A into a phospholipid scramblase as mentioned above (Le et al., 2019b). Interestingly, a previous discovery showed that substitution of a 35 amino acid segment spanning TM4 and TM5 of TMEM16A with the corresponding segment in TMEM16F rendered TMEM16A capable of scrambling phospholipids (Yu et al., 2015). Inspired by the MD simulations of fungal nhTMEM16, a follow-up study identified three additional mutations (V543S, V543T, K588N, numbering based on the TMEM16A(ac) isoform) on two pore lining residues, which can also convert TMEM16A CaCC into lipid scramblases (Jiang et al., 2017). These functional studies thus imply that TMEM16A CaCC may preserve an evolutionary potential to permeate phospholipids. The width of TM4/TM6 separation during gating is likely the key structural determinant for a TMEM16 protein to serve as a pure ion channel or a phospholipid scramblase (Figures 1C,D). For a TMEM16 CaPLSase, Ca^2+^ binding induces wide opening of the TM4/TM6 interface, thereby allowing phospholipid headgroups to gain access and scramble. On the other hand, TM4/TM6 of TMEM16 CaCCs clash with each other in the neck region of the permeation pathway, which prevents them from separating. Therefore, Ca^2+^ binding only allows ion flux without phospholipid permeation. When a charged mutation at the inner gate weakens the interactions between TM4 and TM6, the interface between the two helices may be forced to open widely so that phospholipids can permeate. Future structural, functional, and computational studies are needed to test this hypothesis. It is worth noting that endogenous CaPLSases are ubiquitously expressed in various cell lines (Kunzelmann et al., 2009). Therefore, a cell line without endogenous CaPLSase activity (Le et al., 2019b,c; Liang and Yang, 2021) is essential to experimentally examine the mutational effects on scrambling activities.

## Regulatory Mechanisms of Tmem16 Ion Channels and Lipid Scramblases

### Allosteric Regulation of TMEM16 by a Third Ca^2+^ Binding Site

In addition to the extensively studied primary Ca^2+^ binding sites in TMs 6–8 (Figure 2A), recent structural studies of the mouse TMEM16F and the human ER-localized TMEM16K CaPLSases revealed an additional Ca^2+^ site located in the supporting domain (Alvadia et al., 2019; Bushell et al., 2019; Figure 1B). This third Ca^2+^ site is formed by several charged residues from TM2 and TM10 of the same subunit. In both proteins, the bound third Ca^2+^ ion is coordinated by the carboxylate groups of two highly conserved acidic residues (E395 and D859 in mTMEM16F, E259 and D615 in hTMEM16K) and the main-chain carbonyl group of an isoleucine (I857 in mTMEM16F and I613 in hTMEM16K). The main-chain carbonyl of S854 in TMEM16F (A610 in hTMEM6K) also appears to provide a coordination for the bound Ca^2+^. Interestingly, there is a conserved lysine (K398 in mTMEM16F, K262 in hTMEM16K), which apparently forms a stabilizing electrostatic interaction with the aspartate in TM10.

**Figure 3:**
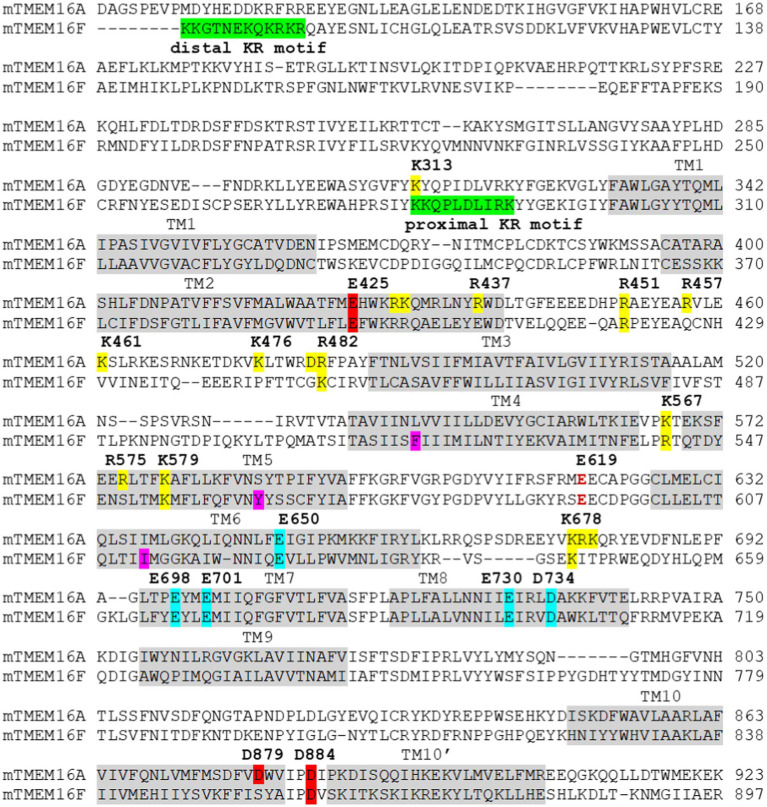
Sequence alignment of the murine(m) TMEM16A (the “a” isoform) and TMEM16F. The transmembrane domains (TM) are highlighted in light gray. The distal and proximal motifs (Aoun et al., 2016; Ye et al., 2018) important for PI(4,5)P_2_ binding in TMEM16F are highlighted in green. Residues that are important for PI(4,5)P_2_ binding in TMEM16A (Le et al., 2019a; Yu et al., 2019; Ko et al., 2020) are highlighted in yellow. Residues at the third Ca^2+^ site (Le and Yang, 2020) are highlighted in red, and residues forming the primary Ca^2+^ sites are highlighted in cyan. Residues that form the inner gate (F518, Y563, and I612) in TMEM16F (Le et al., 2019b) are highlighted in magenta. Intracellular pH affects the primary Ca^2+^ binding sites (cyan highlight; Chun et al., 2015; Liang and Yang, 2021) and extracellular pH works on a conserved glutamate residue (E619 in TMEM16A, dark red text; Cruz-Rangel et al., 2017).

Recently, using the TMEM16A CaCC as a model protein, a comprehensive functional characterization of the third Ca^2+^ binding site in TM2 and TM10 was conducted (Le and Yang, 2020). First, by studying both aWT and the GOF Q645A mutant background, the authors revealed that mutation of the third Ca^2+^ site residues, including E425A, K428A, D879A, and D884A (Figure 3), paradoxically alters channel activation even in the absence of Ca^2+^ binding. Also, because the primary Ca^2+^ sites confound accurate assessment of the third Ca^2+^ site’s function, two charge-reversing mutations, E701K and D734R, both of which eliminate Ca^2+^ binding to the primary Ca^2+^ sites in TMs 6–8, were introduced. The GOF Q645A was included to establish basal channel activity which the authors used to measure the Ca^2+^ sensing capacity of the third site. By eliminating the contribution of the primary Ca^2+^ sites, the authors showed that the third site has a high affinity for Ca^2+^ with an estimated apparent K_D_ of ~320nM, and that Ca^2+^ binding markedly enhances channel activation (Le and Yang, 2020). This hypothesis was bolstered by the observation that single alanine mutations of the three acidic E425, D879, and D884 residues strongly reduce Ca^2+^ sensing of the third site, whereas that of the basic K428 displays a less pronounced reduction. Double alanine mutations of the acidic residues at the third site completely abolish Ca^2+^ sensing, further confirming that the third Ca^2+^ site is solely responsible for the Ca^2+^-dependent activity of the triple mutant background. Strikingly, conformational perturbation of the third site *via* cadmium (Cd^2+^)-mediated bridging of substituted cysteines at E425 in TM2 and D879 in TM10 strongly inhibits channel activation in a manner independent of the primary Ca^2+^ sites. These results could also explain previous studies implicating the functional importance of TM10’, the extended alpha helix following TM10. In fact, replacing or truncating the C-terminal region following TM10 markedly altered the Ca^2+^ sensitivity of TMEM16A (Scudieri et al., 2016; Dang et al., 2017). Chemical crosslinking experiments also suggested that TM10’ may form inter-subunit interactions with the TM2-3 loop (Scudieri et al., 2016), a region that is important for voltage-dependent channel activation (Ferrera et al., 2009; Xiao et al., 2011). Furthermore, the TMEM16K structures also revealed that TM10’ forms inter-subunit interactions with the TM2-TM3 loop and undergoes a pronounced conformational transition during activation of the scrambling pathway (Bushell et al., 2019). Thus, it is tempting to speculate that Ca^2+^ binding to the third site allosterically controls TMEM16A activation, likely by influencing the inter-subunit coupling between TM10’ of one subunit and TM2-TM3 loop of the second subunit (Figure 1B). Future studies are required to fully delineate the functional role and mechanistic underpinnings of the third Ca^2+^ site.

Finally, it is worth noting that several mutations at or near the third Ca^2+^ site have been implicated in several human diseases. A missense mutation of a third Ca^2+^-coordinating residue, D615N, in TMEM16K was identified in a spinocerebellar ataxia type 10 (SCAR10) patient with unknown pathophysiology (Balreira et al., 2014). The equivalent mutation in TMEM16A, D884N, was shown to also reduce channel activation (Le and Yang, 2020). Linkage analysis with exome-sequencing identified 6 pathogenic mutations in TMEM16C that are associated with autosomal-dominant craniocervical dystonia, most notably two missense mutations R494W and W490C (Charlesworth et al., 2012). The W490 and R494 residues are located within TM2 at the putative third Ca^2+^ site flanking the highly conserved K491, which is equivalent to murine TMEM16A K428, murine TMEM16F K398, or TMEM16K K262 (Alvadia et al., 2019; Bushell et al., 2019). A more complete understanding of the third Ca^2+^ site could provide further insight into the human pathophysiological role of these clinically relevant mutations.

### PI(4,5)P_2_-Dependent Regulation of TMEM16 Proteins

Despite constituting only a minor part in the inner leaflet of the plasma membrane, phosphatidylinositol-(4,5)-bisphosphate [or PI(4,5)P_2_] is known to regulate a large number of ion channels and transporters (Suh and Hille, 2008; Hille et al., 2015). PI(4,5)P_2_ was initially suggested to play an inhibitory role in regulating endogenous TMEM16A channels in rat pulmonary artery cells (Pritchard et al., 2014). Reducing PI(4,5)P_2_ levels *via* PLC activation or PI4K inhibition potentiates Ca^2+^-dependent currents of TMEM16A in pulmonary artery smooth muscle cells, whereas addition of PI(4,5)P_2_ markedly reduces its activity. However, it is worth noting that several approaches used to alter PI(4,5)P_2_, namely PLC activation or inhibition of PI4K, could also affect other intracellular signaling events that may lead to changes in intracellular Ca^2+^. One possibility is activation of PLC, while reducing PI(4,5)P_2_ also leads to additional Ca^2+^ release from internal stores, thereby enhancing TMEM16A activation. In fact, numerous subsequent studies from several laboratories all suggested that PI(4,5)P_2_ serves as a positive regulator of TMEM16A (Ta et al., 2017; De Jesus-Perez et al., 2018; Le et al., 2019a; Tembo et al., 2019; Yu et al., 2019; Ko et al., 2020) and paradoxically a negative regulator of TMEM16B CaCC (Ta et al., 2017). Depletion of membrane PI(4,5)P_2_ rapidly desensitizes TMEM16A’s channel activity elicited by sub-micromolar Ca^2+^ both in whole-cell and excised patch recordings. This desensitization under sub-micromolar Ca^2+^ can be rapidly recovered by exogenous application of PI(4,5)P_2_ (Le et al., 2019a; Yu et al., 2019). A hallmark feature of TMEM16 CaCCs is their prominent rundown during prolonged Ca^2+^-dependent activation (Wang and Kotlikoff, 1997; Kuruma and Hartzell, 2000; Ayon et al., 2019; Tembo et al., 2019); exogenous PI(4,5)P_2_ application largely attenuates TMEM16A’s rundown under saturating Ca^2+^ in excised membrane patches (Reisert et al., 2003; De Jesus-Perez et al., 2018; Le et al., 2019a; Tembo et al., 2019).

To gain further insight into the molecular basis of PI(4,5)P_2_-dependent regulation of TMEM16A, unbiased mutagenesis screens were conducted to identify basic residues that play important roles in desensitization in TMEM16A (Le et al., 2019a; Yu et al., 2019). On one hand, Le et al. identified a cluster of basic residues located on the cytosolic sides of TM3, 4, 5, and the TM2-3 loop as the potential binding site for PI(4,5)P_2_ (Figure 3). MD simulations further support spontaneous and favorable PI(4,5)P_2_ binding to this putative site in TMEM16A. Supporting the modular design proposed above, mutating the basic residues in TM3-5 elicits no discernible effects on Ca^2+^-dependent channel gating, despite pronouncedly enhancing current rundown under saturating Ca^2+^ (Figures 1B,C). On the other hand, Yu et al. reported that TMEM16A may harbor a network of PI(4,5)P_2_ binding sites, most notably sites A/1, B/2, and C/4 (Yu et al., 2019). Site A/1 is located near the dimer interface and formed by R429, K430, and R437 of TM2 and K313 of pre-TM1 (Figure 3). Site B/2 is located at the cytosolic C-terminal end of the gating TM6 and mainly consists of K682 (K678 in the (a) isoform), R683 (R679), and K684 (K680; Figure 3). As TM6 and TM7 are both involved in Ca^2+^ binding, PI(4,5)P_2_ binding could directly affect Ca^2+^-dependent channel gating. Finally, site C/4 is situated on TM2-3 loop and is defined by R461 (R457), K480 (K476), and R484 (R480; Figure 3). This site spatially overlaps with the PI(4,5)P_2_ binding site proposed by Le et al., which comprises the TM2-3 linker as well as cytosolic segments of TM3-5 (Le et al., 2019a). MD simulations by Yu et al. also revealed that binding of PI(4,5)P_2_ alters the conformation of the gating TM6 helix, increasing Cl^−^ accessibility, and that occupancy of multiple PI(4,5)P_2_ binding sites led to further dilation of the permeation pathway (Yu et al., 2019).

More recently, Ko et al. reported that TMEM16A exhibits isoform-specific PI(4,5)P_2_ sensitivity (Ko et al., 2020). By co-expressing TMEM16A with the voltage-sensitive lipid phosphatase DrVSP and using whole-cell configuration with 115 or 445nM intracellular Ca^2+^, the authors showed that PI(4,5)P_2_ hydrolysis following membrane depolarization-induced activation of DrVSP led to reduced TMEM16A activity. Interestingly, the TMEM16A(ac) isoform is more sensitive toward PI(4,5)P_2_ depletion than the TMEM16A(a) isoform, which lacks the EAVK segment in the TM2-3 loop. Consistent with the proposed PI(4,5)P_2_ binding site reported by Le et al., Ko et al. also identified R482 (R486 in the TMEM16A(ac) isoform) in TM2-3 loop as the most critical residue for PI(4,5)P_2_ binding (Figure 3). Mutation of R482 to alanine abolishes TMEM16A’s PI(4,5)P_2_ sensitivity, as evidenced by the lack of inhibitory effects on mutant channel activity following PI(4,5)P_2_ degradation by DrVSP. Pharmacological inhibition of CaMKII promotes TMEM16A opening due to augmented single channel conductance. Notably, S669 (S673) at the cytosolic end of TM6 is likely the substrate for CaMKII-mediated phosphorylation, as the phosphomimetic mutation S669D reduces, whereas the S669A mutation enhances the PI(4,5)P_2_ sensitivity of TMEM16A. These results hint at an allosteric mechanism involving PI(4,5)P_2_ binding and CaMKII-dependent phosphorylation in controlling TMEM16A channel activity.

A recent study using multi-microsecond atomistic simulations in explicit solvent and membrane found that specific binding of PI(4,5)P_2_ to the proposed binding site in TM3-5 consistently leads to spontaneous pore opening, which is wide enough to allow Cl^−^ permeation (Jia and Chen, 2021). This pore opening is mediated by the separation of TM4 and TM6 as well as by increased hydration at the central constriction site. It was suggested that upon PI(4,5)P_2_ binding, the cytosolic end of TM4 moves toward PI(4,5)P_2_, whereas its N-terminus (towards the outer leaflet) moves in the opposite direction, thereby separating from TM6 and widening the central constriction site. The “pivot” movement of TM4 is endowed by the helix–helix packing between TM4 and TM5 on the intracellular side. This proposed PI(4,5)P_2_-dependent gating in TMEM16A is reminiscent of the TMEM16 scramblases in which disruption of the TM4 and TM6 interaction leads to opening of the lipid pathway (Figure 1D; Falzone et al., 2019; Kalienkova et al., 2019).

Ion channel activity of TMEM16F also exhibits a reduced Ca^2+^ sensitivity and pronounced current rundown during prolonged Ca^2+^ stimulation, both of which were shown to be a result of the rapid dissociation and/or hydrolysis of endogenous membrane-bound PI(4,5)P_2_ (Ye et al., 2018). Interestingly, an early study on the role of TMEM16F in accessory cholera enterotoxin-stimulated Cl^−^ secretion also suggested that inhibition of PI(4,5)P_2_ synthesis or depletion of PI(4,5)P_2_ markedly attenuated TMEM16F-mediated Cl^−^ current in Caco-2 cells (Aoun et al., 2016). It was suggested that PI(4,5)P_2_ may interact with TMEM16F at two adjacent sites (or KR motifs) at the N-terminus formed by two clusters of basic residues: one proximal site formed by K281-K290 and one distal site formed by K87-R98 (numbering based on the mouse TMEM16F; Aoun et al., 2016; Figure 3). However, whereas mutation or deletion of the distal KR motif did not affect PI(4,5)P_2_ binding, mutation of the basic residues at the proximal KR motif markedly reduced PI(4,5)P_2_ binding, underscoring the functional importance of the proximal KR motif in PI(4,5)P_2_ binding. Paradoxically, electrophysiological studies by Ye et al. (2018) suggested that neutralization of the distal KR motif, including K87, K88, K95, R96, K97, and R98, reduced TMEM16F Ca^2+^ sensitivity as well as the ability of exogenous PI(4,5)P_2_ to rescue TMEM16F current after rundown. By contrast, neutralization of the basic residues in the proximal KR motif (K281, K282, R289, and K290) had no effect on TMEM16F Ca^2+^ sensitivity (Figure 3). While the reason for this discrepancy remains unknown, it could be attributed to their different functional studies—co-IP and electrophysiology–of TMEM16F in addition to the complexity of mutational analyses. Nevertheless, it is worth noting that K313 residue of TMEM16A, which belongs to the equivalent proximal KR motif (K313–K322), could be important for PI(4,5)P_2_ binding, as its mutation significantly reduced the stimulatory effect of PI(4,5)P_2_ on TMEM16A (Yu et al., 2019; Figure 3). So far, no basic residues in TMs 3–5 of TMEM16F, which are equivalent to the proposed regulatory module in TMEM16A (Le et al., 2019a; Yu et al., 2019; Ko et al., 2020), have been implicated in PI(4,5)P_2_ binding. This implies that TMEM16A and TMEM16F may maintain distinct PI(4,5)P_2_-dependent regulation.

A recent structural study revealed the potential structural role of PI(4,5)P_2_ in regulating TMEM16F scrambling (Feng et al., 2019). In the absence of PI(4,5)P_2_, TM6 adopts a straight conformation and PI(4,5)P_2_ supplementation allows it to undergo a pronounced upward movement toward the membrane to widen the intracellular vestibule without changing the ion permeation pore, especially the upper constriction region (Feng et al., 2019). The resulting kinked conformation of TM6 at P628 causes distortion and thinning of the membrane, which is believed to be an important factor for lipid scrambling in TMEM16F (Bethel and Grabe, 2016; Falzone et al., 2019; Kalienkova et al., 2019). Future functional and structural studies are needed to examine if PI(4,5)P_2_ indeed plays a regulatory role in TMEM16F scrambling and whether such PI(4,5)P_2_-dependent conformational changes affect TMEM16F channel activity.

### Intracellular pH Regulation of TMEM16 Proteins

Previous studies showed that low intracellular pH (pH_i_) suppresses endogenous Ca^2+^-activated Cl^−^ channels (CaCCs) from the human colon carcinoma cell line T84 and lacrimal gland acinar cells (Arreola et al., 1995; Park and Brown, 1995). Consistent with these observations, low pH_i_ was shown to strongly inhibit channel activation of heterologously expressed TMEM16A, TMEM16B, and TMEM16F ion channel activity (Chun et al., 2015). Low pH_i_ causes a rightward shift in the Ca^2+^ EC_50_ curves of TMEM16A and TMEM16B without affecting the voltage-dependent, heat-dependent, or E_act_-mediated (E_act_ is a putative activator of TMEM16A) activation of TMEM16A. The authors further demonstrated that double mutation of Ca^2+^ binding residues in TM6-8, including N650A/E654Q (TM6, numbering based on the TMEM16A(ac) isoform), E702Q/E705Q (TM7), and E734Q/D738N (TM8) abolished this proton-mediated inhibition. Based on this evidence, the authors proposed that protons may inhibit TMEM16A channel activation by competing with Ca^2+^ binding to Ca^2+^ binding sites in TM6-8.

A recent comprehensive investigation of pH_i_ regulation on TMEM16 proteins, including TMEM16A ion channel activity and TMEM16F ion channel and lipid scrambling activities, was conducted using a patch clamp-lipid scrambling fluorometry (PCLSF) assay (Liang and Yang, 2021). Consistent with previous results in HEK293 cells (Chun et al., 2015) and in native cells (Arreola et al., 1995; Park and Brown, 1995), low pH_i_ was found to significantly attenuate TMEM16A and TMEM16F ion channel activities and TMEM16F lipid scrambling activity. In addition, high pH_i_ largely potentiates TMEM16A and TMEM16F ion channel activities and TMEM16F-lipid scrambling activity. Mechanistically, pH_i_ exerts its effect specifically on the two primary Ca^2+^ binding sites, as evidenced by the following results. First, the binding site point mutation E667Q significantly suppresses intracellular pH sensitivity of TMEM16F ion channel activity, consistent with previous results (Chun et al., 2015). Second, pH_i_ exerts negligible effects on the pore-lining residue, Q559K, and the third Ca^2+^ binding site, D859A and E395A. Third, pH_i_ exerts no effect in the absence of intracellular Ca^2+^ on GOF mutations, namely TMEM16A L543Q and Q645A and TMEM16F Y563K and F518K. Based on these observations, pH_i_ regulatory effects were proposed to stem from protonation or deprotonation of the Ca^2+^ binding sites, which in turn reduces or enhances Ca^2+^ binding affinity, respectively. Identifying the molecular underpinning of pH_i_ regulation of TMEM16 ion channel and scrambling activities will help contextualize their physiological and pathological roles, such as in platelet activation, tumor progression, and sperm–egg fusion (Whitlock, 2021).

### Extracellular pH Regulation of TMEM16 Proteins

In contrast to the effects by pH_i_ on TMEM16A, low extracellular pH enhances TMEM16A channel opening without altering the apparent Ca^2+^ sensitivity (Cruz-Rangel et al., 2017). This suggests that extracellular pH does not exert its effect through the Ca^2+^ binding sites like pH_i_. Using mutagenesis screening of the extracellular acidic residues, the authors found that one residue, E623, located at the extracellular end of TM6, largely suppresses the effect of extracellular pH on TMEM16A when mutated to alanine. They suggested that protons likely function by promoting protonation of E623, which reduces the energy barrier for Cl^−^ entry. It should be noted that E623 (E619 in the (a) isoform) of TM6 and R515 (R511) together constitute the equivalent SE site proposed by Bethel and Grabe (2016). As this residue is highly conserved in all the TMEM16 family proteins, it is likely that extracellular pH also influences other TMEM16 members, including TMEM16F. Future investigations will be needed to assess the effects of extracellular pH on other TMEM16 members.

## Future Perspectives

Structural, functional, and computational studies in the past decade have greatly advanced our understanding of TMEM16 proteins at the molecular level. In the next phase, the answers to the following questions will further advance our understanding of these enigmatic proteins. First, it will be important to demonstrate how the third Ca^2+^ site is allosterically coupled to the PGD and how all three Ca^2+^ bindings sites synergistically control TMEM16 activation under physiological conditions. Second, future investigations are needed to dissect how Ca^2+^ and voltage synergistically operate TMEM16 gating. The answer to this question is critical to uncover the physiological functions of TMEM16 proteins in excitable cells such as neurons and muscles. Together, we have started to understand the molecular mechanisms of TMEM16 ion and lipid permeation and identified several molecular determinants that define whether a TMEM16 protein is a sole ion channel or a dual function scramblase/ion channel. Comprehensive studies are needed to demonstrate how ion and phospholipid permeation are dynamically controlled by Ca^2+^- and voltage-induced conformational changes in the PGD. Substantial progress has been made on deciphering how pH and PI(4,5)P_2_ regulate TMEM16 proteins. Identifying other physiological regulatory factors, such as post-translational modifications, are needed to further reveal how TMEM16 protein activities are fine-tuned under physiological conditions. Additionally, the Ca^2+^-bound TMEM16A and TMEM16F structures were captured in non-conductive states. Future structural studies are needed to capture the open conformations, which will enhance our understanding of TMEM16 gating transitions in response to Ca^2+^ and voltage stimulation. Apart from the ER-resident TMEM16K, the other mammalian TMEM16 proteins expressed in intracellular organelles are largely uncharacterized. Functional and structural characterization of these TMEM16 proteins will help us better evaluate their biological functions in health and disease. Finally, the evolutionary relationships between TMEM16, OSCA/TMEM63, and TMC proteins within the transmembrane channel-scramblase (TCS) superfamily are intriguing. A combination of structural, functional, and computational approaches is needed to unveil the molecular underpinnings of how this superfamily of membrane ion channels and scramblases posses different permeation, activation, and gating properties. In this review, we summarize the collective efforts from the TMEM16 field over the past decade. We propose a “modular design” model for TMEM16 assembly, and the “clam-shell” and “pore-dilation” gating/permeation models for TMEM16 scramblases and channels, respectively. We hope these simplified models serve as a steppingstone for answering the aforementioned questions, driving the field forward.

## Author Contributions

SCL, PL, AJL, and HY wrote the manuscript. SCL plotted all the figures. All authors contributed to the article and approved the submitted version.

## Funding

This work was supported by NIH grant DP2-GM126898 (to HY) and American Heart Association Pre-doctoral Fellowship 19PRE34380456 (to SCL).

## Conflict of Interest

The authors declare that the research was conducted in the absence of any commercial or financial relationships that could be construed as a potential conflict of interest.

## Publisher’s Note

All claims expressed in this article are solely those of the authors and do not necessarily represent those of their affiliated organizations, or those of the publisher, the editors and the reviewers. Any product that may be evaluated in this article, or claim that may be made by its manufacturer, is not guaranteed or endorsed by the publisher.
